# Celebrating 150 volumes of *Parasitology* with an outlook towards 2030 production

**DOI:** 10.1017/S0031182024000167

**Published:** 2024-03

**Authors:** J. T. Ellis, J. R. Stothard

**Affiliations:** 1 School of Life Sciences, University of Technology Sydney, Ultimo, NSW 2007, Australia; 2 Department of Tropical Disease Biology, Liverpool School of Tropical Medicine, Liverpool L3 5QA, UK

## Abstract

**Figure:**
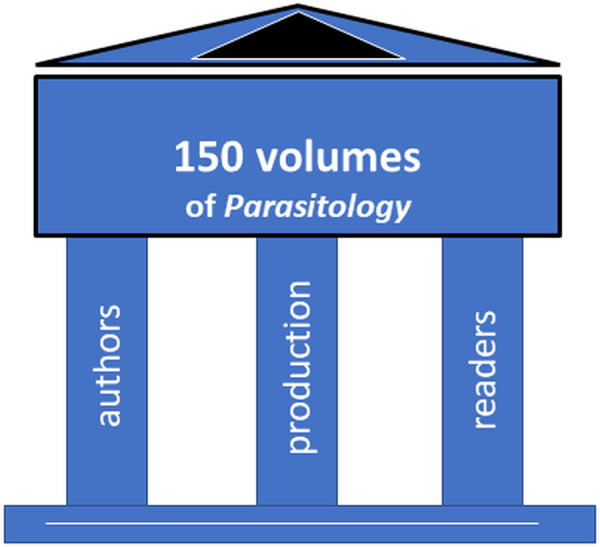

Avid readers of our journal will have noticed that the end of 2023 saw the completion of the 150th volume of *Parasitology*. This is a landmark statistic in long-term scientific publishing which defines an impressive academic legacy that goes on to support the future study of parasites around the world. Since January 2023, *Parasitology* has been following a Gold Open Access Model, specifically a non-exclusive Gold Open Access CC-BY licence (see https://www.cambridge.org/core/services/open-access-policies/open-access-resources/creative-commons-licenses).

As Cambridge University Press expected, this OA transition was non-trivial and required detailed planning, beginning in 2020, using a theory-of-change (ToC) model to underpin strategic engagements across the various stakeholders of our journal (Stothard, Ainsworth and Marriott, 2021). Behind this OA transition have been the unchanged contributions and continued hard work of the journal's referees. We thank them most sincerely (List of reviewers in 2023, 2024), for without their *pro bono* services, *Parasitology*'s scientific rigour and reputation would decline.

In this year's editorial, we elaborate on the implications of our recent OA transition. Looking ahead to production in 2030, we provide a short synopsis of several issues to address and to develop.

## Open access agreements and responsibilities

Cambridge University Press maintains a database of over 2000 academic institutes eligible within the Transformative Agreements for *Parasitology* (see https://www.cambridge.org/core/services/open-access-policies/read-and-publish-agreements). There is a convenient online checker (see https://www.cambridge.org/core/services/open-access-policies/waivers-discounts) and if your institute is not presently included, some local canvassing within your academic institute may be needed to join. For more informed discussions, useful information is provided here (see https://www.cambridge.org/core/services/open-research/open-access/open-access-journal-flips). *Parasitology* also has a very generous waiver programme, to enable all authors to publish in the journal regardless of funding, including a discretionary waiver option for authors who do not have funding and are not eligible for the Research4Life scheme. To apply for a discretionary waiver, authors only need to complete this form (see Waiver or Discount Request: Cambridge University Press (PS) (office.com) with a short letter from their institution confirming no current funding. A waiver will be later granted.

## Encouraging global access and impact with OA

While parasites can be found across all seven continents, the distribution of parasitologists active or those interested in parasitological research is less geographically diverse. Even though a seminal ambition of OA publishing was to provide unrestricted access to scientific articles, from anywhere in the world, *Parasitology* remains mindful that the geographical balance between authors and readers needs regular monitoring. This is to flag any emerging imbalance that can be addressed by corrective measures. Like our editorial board's composition, foremost, we aim to ensure an equitable geographical representation within our current and future authorship, alongside promotion of better gender balance as much as possible. To do so, a new initiative of *Parasitology* has been to support the activities of societies outside of the UK. A good example was the production of our Special Issue dedicated to specific outputs from the Italian Society for Parasitology that showcased several presentations made within their symposium at Naples (Cassini *et al*., 2023).

Alternatively, for those parasitologists unable to attend a face-to-face symposium, *Parasitology* successfully completed an international commissioned Special Issue themed on Avian Malaria (Ranford-Cartwright, 2023). The latter strategy, with its subsequent scientific theme is elaborated below. There is to be a growing list of future synergistic activities, for example, those to be set within the 21st International Congress for Tropical Medicine and Malaria (ICTMM) (see https://ictmm2024.org/). This important congress is hosted by the Malaysian Society for Parasitology and Tropical Medicine (MSPTM). It is locally led by Professor Dr Siti Nursheena Mohd Zain, who is a member of our editorial board (see https://www.cambridge.org/core/journals/parasitology/information/about-this-journal/editorial-board).

**Figure 1.**
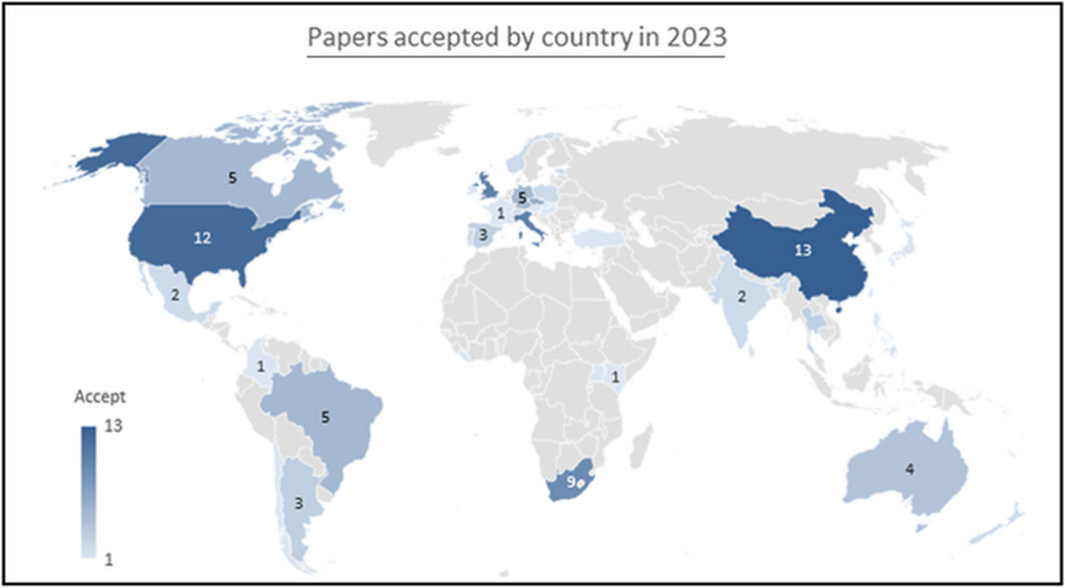
The global geographical fingerprint of authors from papers published in *Parasitology* in 2023, (our first year of being fully OA). While USA, UK and Australia have been location strongholds of authors, it is very pleasing to see increasing submissions from Africa and Asia. To broaden the immediate appeal of *Parasitology* outside of Anglophone restrictions, we are considering future ways to abstract our articles in alternative international languages. We are also exploring ways to encourage female researchers, at whatever stage of their career to submit their articles for consideration.

As shown in Fig. 1, we are proud to report that during the last year our global geographical fingerprint of authors expanded although there is more to be done to continue to inform, encourage and promote our authors. Indeed, we hope to persuade potential authors that submission of their article to *Parasitology* is a rewarding experience both from short- and long-term perspectives.

## Strengthening website and social media portals

In 2022, *Parasitology* ceased hardcopy production with all articles produced and posted online only (Stothard and Ellis, 2022). To strengthen our website and communications portfolio, we expanded our online offer with enhanced social media activities. Since the introduction of our ‘*Paper-of-the-Month*’ blog, this social media feature has grown in importance to help the journal promote a key article each month. These articles are selected by one of our editors after catching their interest, with our social media team commissioning a digest blog from the authors.

**Figure 2.**
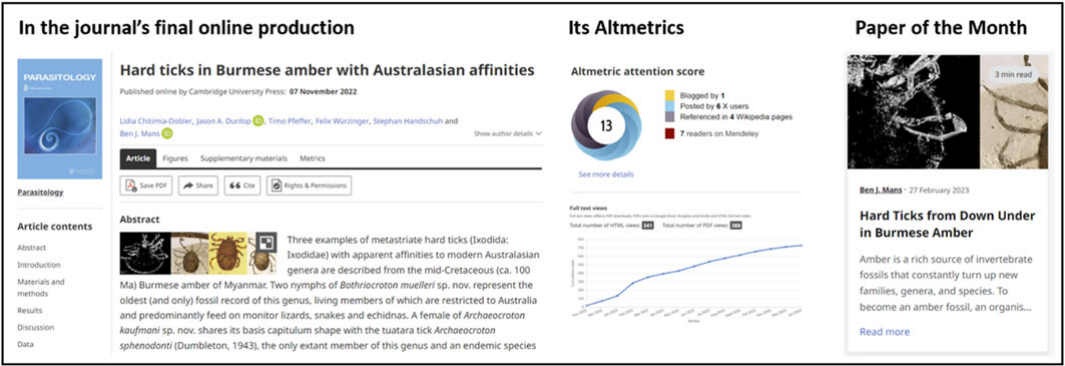
A social media highlight of *Parasitology* in 2023 during our first year of being fully OA. This article on Burmese ticks in Amber was selected as Paper-of-the-Month with associated promotion *via* social media. We welcome other short blogs that provide narratives on our articles’ impact.

Most recently we have recruited Dr Joel Barratt, CDC Atlanta, USA to join the social media editorial team. It is very pleasing indeed to see well over 30 articles now posted, each giving an alternative insight into the findings and implication of the selected ‘*Paper-of-the-Month*’. A good example is provided by Fig. 2, drawing attention to the evolutionary importance of fossil ticks in amber. To enhance our blog offer, we openly welcome other short articles that provide a narrative on articles' impact. If you have a topic in mind, please contact the editorial office with your suggestion.

An influential annual activity engaging with our authorship and readership is our front cover image competition, won this year by Andrea Langeland (see https://www.cambridge.org/core/blog/2023/12/07/our-new-front-cover-image-for-parasitology-2024-by-andrea-langeland/). Incidentally, Andrea's research also featured in an earlier ‘*Paper-of-the-Month*’ article (see https://www.cambridge.org/core/blog/2023/06/16/comparative-transcriptomics-from-intestinal-cells-of-permissive-and-non-permissive-hosts-during-ancylostoma-ceylanicum-infection-reveals-unique-signatures-of-protection-and-host-specificity/). We can therefore safely assume that readers of *Parasitology* are now much better aware of the zoonotic importance of *Ancylostoma ceylanicum* and appreciate her efforts to identify this parasite's differentially expressed genes.

We are proud to support the Early Career Researcher (ECR) Award and Irish Society for Parasitology William C. Campbell Prize each year. The annual Irish Society meeting is dedicated to the promotion and training of ECRs (including PhD students), which results in high-quality presentations meriting Cambridge University Press support. As authors (and readers) realize, the use of social media is ever more important in raising general awareness of their articles. This is primarily to amplify their communication and research uptake impacts. Other activities include the promotion of our virtual collections, for example, the clutch of articles themed on ‘*Parasites detected by eDNA and metabarcoding methods*’, (see https://www.cambridge.org/core/journals/parasitology/collections/parasites-detected-by-edna-and-metabarcoding-methods). Looking ahead to the latter half of 2025, we seek to commission an international Special Issue on these fast-moving technologies within Life and Environmental Sciences, see below.

## Securing pertinent themes for our special issues

*Parasitology* has an unrivalled history of producing Special Issues that explore or focus attention on a specific aspect of research. Since the remit of *Parasitology* is broad, our diversity of themes across the last five years has been impressive, featuring many dimensions within the biology of worms and protists. A highlight was our support and production of nematode research originating from the ‘*6th International Workshop on Angiostrongylus and Angiostrongyliasis, Hilo, Hawai'i, USA, January, 2020*’. This brought together 17 articles, with an overarching editorial preface (Jarvi and Prociv, 2021), and explored the use of a short video interview for Professor Sue Jarvi. Sue explained the Special Issue's context and global importance within wider biomedical research (see https://youtu.be/xtLAAA6lXoQ).

In keeping with ambitions to further engage internationally with the scientific community and to expand our readership and authorship base, *Parasitology* is collaborating with the MSPTM on the 2024 ICTMM, to be held in Sarawak, Malaysia. With Cambridge University Press sponsorship of the ICTMM and with formal collaborations with MSPTM, two special issues of *Parasitology* are each expected, one on malaria in primates and the other on parasites of the genital tract. Collaboration with MSPTM also includes editorial support for their journal, *Tropical Biomedicine*. For example, they will seek to commission manuscripts on ticks and tick-borne diseases which springboards from our current open call for manuscripts for ticks and tick-borne diseases, led by Professor Ala Tabor (University of Queensland), but with a greater focus on Southeast Asia.

On the international front, we highlight the importance of contributions from our colleagues in South America. As a result of regular reviews of submissions worldwide, we have documented the difficulties that have arisen in South America owing to the flip to OA publishing. To reward, support and encourage submissions from South America further, two concepts for special issues are under development; Professor Vyacheslav Yurchenko (University of Ostrava) volunteered to lead a special issue in the general area of Haemoflagellates, while Dr Carolina De Marco Verissimo (University of Galway) is pursuing a call on zoonotic diseases with a focus on ECR authors. We look forward to seeing these concepts through to fruition.

The use of ‘open-calls’ by other scientific journals is now widespread. It is, however, perceived by some as tiresome owing to a proliferation and excessive use of advertising. To retain our competitiveness, *Parasitology* has introduced a carefully controlled process commencing with our Avian Malaria theme. By limiting ‘open-calls’ to a small number of specialist topics, each selected and agreed upon by considerations of the editors and editorial board, this helps to maintain our unique standing within the discipline of parasitology. So please fear not(!), we will not over burden our respected and distinguished community with un-warranted spam and repetitive solicitations. We do of course encourage potential authors to check regularly the journal's homepage for forthcoming Special Issues and our e-collections; if any member believes very strongly that a parasitology topic should be covered, we encourage you to contact our editors directly.

Cambridge University Press is always considering novel ways to best highlight and promote the incredible research performed by our authorship, alongside encouraging fresh dialogue with our readership. One such initiative is our plan to hold a one-day symposium at Cambridge University to highlight the fascinating biology of parasites within the genital tract of livestock and wildlife, augmenting our ICTMM symposium. This will be led by Professor Cinzia Cantacessi (Cambridge University). Please inspect our homepage for updates later this year.

## Towards 2030 production

The current United Nations Sustainable Development Goals are framed within a 2015–2030 vista; it is therefore appropriate to consider what further changes are needed to ensure *Parasitology* remains a ‘go-to’ publishing service and stays successful within the Cambridge University Press scientific portfolio. Of note, we are following recommended ways to keep the production of *Parasitology* as environmentally friendly as possible. To do so, we follow our ToC model, mindful of the many inputs and outputs from a network of both external and internal stakeholders. This is regularly discussed during online meetings of the editors and annually at our face-to-face Editorial Board Meeting. The latter forum has online participation from our full editorial board.

While *Parasitology* will continue to publish in English, to broaden our immediate appeal outside of anglophone restrictions, we are considering future ways to abstract our articles in alternative international languages. This will go some way to help with online searching and citation of information within systematic reviews, especially for those, for example, who do not require publication in English. To broaden *Parasitology*'s appeal, we intend to expand upon the use of our graphical abstracts with the future inclusion of short videos to embellish key papers. This use of multimedia is to foster interactions with other scientists, international societies and agencies aligned with our journal's objectives. To promote our articles further, we are developing better integration of an article's key findings with messaging on our social media platforms. Please remember to subscribe to our X (formerly Twitter) account @JnlParasitology and our Facebook page (see https://www.facebook.com/JnlParasitology/) to keep better informed.

To close, we look forward to the opportunities that 2030 will bring, knowing that the existence of *Parasitology* is to serve this academic discipline. We strive to support all who take part in, or make use of, carefully gained knowledge assembled in *Parasitology*. This features parasites and parasitism across the seven continents of the world. Our fully OA model now better enables us to be fully global in our impact.

## Editors

Russell Stothard, *Editor-in-Chief*, 2020 –

John Ellis, *Deputy Editor-in-Chief & Special Issues Editor*, 2021 –

Cinzia Cantacessi, 2022 –

Joseph Jackson, 2022 –

Laura Rinaldi, 2020 –

Lisa Ranford-Cartwright, 2021 –

Jonathan Wastling, 2011 –

## Social media editors

Emily Pascoe, *Senior Social Media Editor*, 2022 –

Joel Barratt, 2023 –

Derrick Osakunor, 2022 –

Maureen Williams, 2019–2022

## Data Availability

Not applicable.
